# Characterization of Caulimovirid-like Sequences from Upland Cotton (*Gossypium hirsutum* L.) Exhibiting Terminal Abortion in Georgia, USA

**DOI:** 10.3390/v16071111

**Published:** 2024-07-11

**Authors:** Surendra R. Edula, Lavesta C. Hand, Phillip M. Roberts, Edward Beasley, John L. Snider, Robert C. Kemerait, Peng W. Chee, Sudeep Bag

**Affiliations:** 1 Department of Plant Pathology, University of Georgia, Tifton, GA 31793, USA; 2 Department of Crop and Soil Sciences, University of Georgia, Tifton, GA 31793, USA; 3 Department of Entomology, University of Georgia, Tifton, GA 31793, USA; 4 Institute of Plant Breeding, Genetics, and Genomics, University of Georgia, Tifton, GA 31793, USA

**Keywords:** endogenous viral elements, terminal abortion, upland cotton, lncRNA, small RNA

## Abstract

In this study, we investigated the potential involvement of endogenous viral elements (EVEs) in the development of apical tissue necrosis, resulting in the terminal abortion of upland cotton (*Gossypium hirsutum* L.) in Georgia. The high-throughput sequence analysis of symptomatic and asymptomatic plant tissue samples revealed near-complete EVE-Georgia (EVE-GA) sequences closely related to caulimoviruses. The analysis of EVE-GA’s putative open reading frames (ORFs) compared to cotton virus A and endogenous cotton pararetroviral elements (eCPRVE) revealed their similarity in putative ORFs 1–4. However, in the ORF 5 and ORF 6 encoding putative coat protein and reverse transcriptase, respectively, the sequences from EVE-GA have stop codons similar to eCPRVE sequences from Mississippi. In silico mining of the cotton genome database using EVE-GA as a query uncovered near-complete viral sequence insertions in the genomes of *G. hirsutum* species (~7 kb) but partial in *G. tomentosum* (~5.3 kb) and *G. mustelinum* (~5.1 kb) species. Furthermore, cotton EVEs’ episomal forms and messenger RNA (mRNA) transcripts were detected in both symptomatic and asymptomatic plants collected from cotton fields. No significant yield difference was observed between symptomatic and asymptomatic plants of the two varieties evaluated in the experimental plot. Additionally, EVEs were also detected in cotton seeds and seedlings. This study emphasizes the need for future research on EVE sequences, their coding capacity, and any potential role in host immunity or pathogenicity.

## 1. Introduction

Endogenous viral elements (EVEs) were discovered within the genomes of numerous protists [1,2,3], fungi [4], insects [5,6,7,8], fish [9], birds [10,11], plants [12], animals [13,14], humans [15,16,17], and other mammals [18]. These are considered relics of ancient infections, occasionally referred to as genomic fossils. The first EVEs were discovered in the late 1960s, and much before the discovery of reverse transcriptase, they were endogenous retroviruses, otherwise called endogenous retrovirus elements [19]. They integrate into the host nuclear genome with the help of ‘integrase’, a virally encoded recombinase that catalyzes the viral DNA insertion into the host cell’s DNA [19,20]. Among plants, EVEs were first discovered in the tobacco (*Nicotiana tabacum*) genome with geminivirus-related DNA (GRD) [12,21], followed by the caulimovirid (members of the family *Caulimoviridae*)-like DNA in the tobacco (*N. tabacum*) and banana (*Musa balbisiana*) genomes [22,23]. The GRD found in tobacco originated from an ancient New World geminivirus integration believed to be an illegitimate recombination event in a New World *N. tabacum* [12]. Such a similar phenomenon (non-homologous DNA end-joining) must have endogenized the partial or near-complete sequences of the members belonging to the family *Caulimoviridae* during the host meiotic (crossovers) or mitotic recombination (somatic DNA repair) during exogenous ancient infections [24,25]. Such integrations in host germline cell chromosomes can lead to vertical transmission and are further inherited as host alleles [14,26]. Endogenous viral elements represent the DNA sequences of viruses that integrate into the host genome and are capable of vertical transmission. Viral DNA integration into bacterial and animal host chromosomes is a common phenomenon. Such integrations in plant genomes are still under study [27]. The most described EVEs are from viruses with DNA genomes (ssDNA, genus: *Geminivirus* and dsDNA, genus: *Caulimovirus*) or viruses that can exhibit DNA phases in their replication.

In plants, the frequently observed EVEs, called “plant pararetroviruses”, belong to the genus *Caulimovirus*, and their sequences are believed to be embedded into their host genomes due to ancient virus infections dating back thousands to millions of years ago [28]. The family *Caulimoviridae* (order *Ortervirales*) [29] comprises 11 genera, including the following: *Badnavirus*, *Caulimovirus*, *Cavemovirus*, *Dioscovirus*, *Petuvirus*, *Rosadnavirus*, *Ruflodivirus*, *Solendovirus*, *Soymovirus*, *Tungrovirus*, and *Vaccinivirus* [30]. The members of this family consist of either isometric or bacilliform-shaped non-enveloped virions and comprise reverse-transcribing non-covalently closed circular dsDNA genomes of 7.1–9.8 kbp in size, which replicate through RNA intermediates. Depending on the genus, members of the family *Caulimoviridae* may comprise one to nine open reading frames (ORFs) in their genome. Despite their diversity, all members code for various common proteins, including a movement protein, a coat protein, a pepsin-like aspartic protease, reverse transcriptase (RTase) with a bound RNase H1, and several genera also code for transactivator/viroplasm protein [30,31]. Genome replication of the *Caulimoviridae* family members is performed by reverse transcription, which is similar to retroviruses but without integration into the host DNA. Further, transcription of terminally redundant RNA of caulimovirus is performed by episomal DNA mini chromosomes using host RNA polymerase II in the infected nuclei [32,33]. Caulimoviruses encode a polycistronic mRNA, and the expression is through ribosomal reinitiation and translational coupling of individual genes [33,34].

Among the existing 11 members of the family *Caulimoviridae*, EVEs were reported from *Badnavirus*, *Cavemovirus*, *Caulimovirus*, *Petuvirus*, and *Tungrovirus* [22,35,36,37,38]. Caulimovirus EVEs occurrence was discovered in many vascular monocots, dicots, and even ferns [23,39,40,41,42,43,44]. Most of the integrated EVEs are replication-defective and distributed in varied sizes and numbers among their host genomes, ranging from shorter fragments to near-complete virus sequences [25]. Until recently, such EVEs were not discovered in the cotton genome.

Upland cotton (*Gossypium hirsutum* L.) is extensively cultivated worldwide, primarily for its fiber. However, it is susceptible to a range of pathogens and pests, including plant viruses (geminiviruses, ilarviruses, and poleroviruses) and their insect vectors (whiteflies, thrips, and aphids) [45,46,47]. In early June of 2023, upland cotton in Georgia (GA), USA, experienced both abiotic and biotic challenges. This period was marked by cooler-than-average summer temperatures alongside above-average infestations of tarnished plant bugs (TPB; *Lygus lineolaris* Palisot de Beauvois), as reported in the UGA extension newsletter, June 2023 (http://www.ugacotton.com/newsletter/) [Accessed on 25 May 2024]. Along with these issues, it was noted that young cotton plants frequently exhibited terminal abortion, resulting in the development of profuse vegetative branching. The cause of these symptoms could not be attributed to the other issues occurring in the 2023 growing season. Affected young cotton plants exhibiting terminal abortion developed two or more vegetative branches, with major occurrences noted in plants at the 3-leaf stage and a few instances at the 5–6-leaf stage.

Various thrips species serve as vectors for transmitting plant viruses, including *Orthotospovirus tomatomaculae* (formally known as the tomato spotted wilt virus, TSWV) in crops like peanut, pepper, and tomato [48,49] and tobacco streak virus (TSV) in cotton [46]. TSV is widespread in cotton-growing countries worldwide [46], and it has a broad host range [50,51,52,53,54,55]. While TSV has not been documented in cotton in the USA, its presence has been reported in yellow summer squash (*Cucurbita pepo* L.) in GA, USA [54].

Initially, the early infestation of thrips on emerging cotton led to the hypothesis that thrips transmitted TSV as a potential pathogen for necrosis and terminal abortion. Additionally, another suspected viral pathogen was cotton leafroll dwarf virus (CLRDV), which is prevalent in Georgia and other cotton-growing regions in the USA [47]. Recent discoveries of endogenous cotton pararetroviral elements (eCPRVE) sequences [25] and a novel cotton virus A (CotV-A) [56] in cotton from Mississippi prompted us to investigate the presence of such viruses and viral elements in the field samples in GA. With the advancement of sequencing technologies, high-throughput sequencing (HTS) of small RNA [57,58] and lncRNA [25] is widely used for the detection of known and novel viruses without any prior knowledge [59]. Further, this study aims (i) to characterize EVEs within the cotton genome using HTS and to conduct in silico assessment of cotton (*Gossypium* species) genomes for endogenous caulimovirid-like sequences using cotton genomic databases, namely Phytozome (https://phytozome-next.jgi.doe.gov) [Accessed on 25 May 2024] [60] and Cottongen (https://www.cottongen.org/) [Accessed on 25 May 2024] [61], (ii) to evaluate the presence of their episomal forms and mRNA transcripts of movement protein gene, in addition to investigating for unknown or cryptic viruses in the small RNA sequences [62,63] extracted from field samples.

## 2. Materials and Methods

### 2.1. Sample Collection

In early June of 2023, during the vegetative stage of the crop, cotton plants exhibiting terminal abortion (symptomatic) and plants devoid of such symptoms (asymptomatic) (*n* = 54) were collected from commercial cotton fields in two locations: the Sunbelt Agricultural Expo (*n* = 28) in Colquitt County and Hopeful (*n* = 26) in Mitchell County, GA. The tissue sample (petiole, leaf, and tissue near the vegetative branching) from each plant was combined and processed to diagnose the presence of potential viruses impacting cotton at the Virology lab, UGA Tifton, GA. Samples from Colquitt County were pooled into three subsamples: symptomatic S1 (*n* = 12), S2 (*n* = 12), and asymptomatic S3 (*n* = 4). Similarly, samples from Hopeful were pooled into three subsamples: symptomatic S4 (*n* = 11), S5 (*n* = 11), and asymptomatic S6 (*n* = 4), resulting in six composite samples.

An experimental plot was established at the UGA Bowen research farm in Tifton, GA, to evaluate the incidence of terminal abortion and its potential impact on yield. Two varieties of cotton, ‘Dyna-Gro 3615 B3XF’ and ‘Dyna-Gro H959 B3XF’ (DG3615 and DGH959, Loveland Products, Inc., Loveland, CO, USA), were planted in individual plots, which were 16 rows wide and 750 feet in length. The four middle rows were selected for each variety to evaluate the association of EVEs with the terminal abortion observed in the commercial fields, excluding the four border rows to avoid border effects. During the vegetative growth phase for both varieties, DG3615 and DGH959, a symptomatic plant was selected in each row and marked along with an adjacent asymptomatic plant. This was replicated five times in each row, and there were twenty replications for each variety. For DGH959 only, one adjacent asymptomatic plant was additionally selected, and the apical bud was manually terminated to induce terminal abortion. Twenty symptomatic and twenty asymptomatic plants were selected for each variety, and an additional twenty induced terminal abortion plants were selected in DGH959 only. A total of 100 individual plant samples were labeled/tagged and monitored for symptom development and progress. Yield data were hand-harvested from individual plants.

### 2.2. Statistical Analysis

For DG3615, symptomatic and asymptomatic plants are considered two treatments, and a paired student’s *t*-test was performed to determine the effect of treatments on the seedcotton yield plant^−1^ and the boll density plant^−1^. There were three treatments (symptomatic, asymptomatic, and induced terminal abortion plants) in DGH959, and a one-way mixed effects analysis of variance (ANOVA) was used to determine the effects of treatments on the same response variables. Significant effects of treatments were considered at *p* < 0.05. Statistical analysis was performed using JMP Pro version 16 (SAS Institute; Cary, NC, USA).

### 2.3. Seed and Seedling Assessment

**Figure 1:**
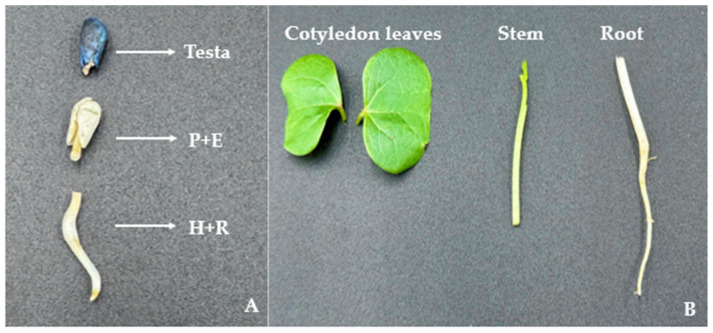
Cotton seed and seedlings testing for endogenous viral element (EVE) detection. (**A**) Cottonseed parts (testa, plumule with epicotyl, and hypocotyl with root tissue) and (**B**) cotton seedling parts (cotyledon leaves, stem, and root tissue).

Seeds and seedlings from four different commercial cotton varieties, Deltapine 1646 B2XF (DP1646, Bayer Crop Science, St. Louis, MO, USA), Stoneville 4595 B3XF (ST4595, BASF Corporation, Research Triangle Park, NC, USA), DG3615, and DGH959, were evaluated in both laboratory and greenhouse settings. This evaluation was aimed at ascertaining whether the EVE’s expression was induced in field conditions due to abiotic factors or was also evident in controlled environments. Ten seeds from each variety were sown directly in soil (Pro-Mix Premier HP, peat-based growing medium) using 6-well trays of 1.5″ square by 2.25″ depth and were maintained inside the insect-free cages (BugDorm, 160 µm aperture, MegaView Science Co., Ltd. Taichung, Taiwan, China) in the greenhouse facility at the University of Georgia, Tifton, GA. The greenhouse was maintained at a temperature of 28 ± 3 °C and 50 ± 20% relative humidity throughout the experiment. Simultaneously, the other set of ten seeds from each variety were kept on water-soaked filter paper in a petri dish and incubated at room temperature under dark conditions to induce seed germination. All varieties except DP1646 were available pre-treated with fungicide. After 96 h, sprouted seeds and seedlings were divided into three parts for EVEs testing. The seed coat (testa) was separated, and the sprouted seed was divided into the upper shoot with plumule and epicotyl (P + E) and lower root parts, including the hypocotyl, and root (H + R) (Figure 1A). Meanwhile, the seedlings grown under greenhouse conditions were collected, and each seedling was separated into three different parts for EVE analysis (cotyledon leaves, an inch of stem including the meristem, and root parts) (Figure 1B).

### 2.4. Nucleic Acid Extraction (DNA, RNA, and TNA)

The total RNA was extracted from the six commercial composite field samples from Colquitt and Mitchell County using Spectrum^TM^ Plant Total RNA extraction kit (Sigma–Aldrich, St Louis, MO, USA) following the manufacturer’s protocol. The total DNA was extracted from the same set of samples using the DNeasy Plant Mini Kit (Qiagen, Germantown, MD, USA). Extracted RNA and DNA were used for PCR assays, and the total RNA was used for library preparation and high-throughput sequencing.

Additionally, total nucleic acid (TNA) was extracted using magnetic bead technology following the protocol as described in Adeleke et al. [63] from individual field samples collected from Colquitt (*n* = 28) and Mitchell (*n* = 26) counties, leaf petiole samples (*n* = 100) collected from UGA Bowen farm experimental plot, and seeds (*n* = 50) and seedlings (*n* = 50) from the greenhouse and laboratory.

The quality and quantity of the total nucleic acids were determined using a NanoDrop One UV–Vis Spectrophotometer (Thermo Fisher Scientific, Waltham, MA, USA). DNA, RNA, and TNA, with 260/280 absorbance of ≥1.8, were aliquoted for further analysis and stored at −80 °C.

### 2.5. Nucleic Acid Treatment

To detect mRNA transcripts of EVEs and to eliminate erroneous detection of the integrated cotton EVE sequences in the plant genomic DNA, we treated four commercial cotton field composite RNA (S2: symptomatic; S3: asymptomatic; S5: symptomatic; and S6: asymptomatic) and TNA extracted samples with DnaseI (Thermofisher, Waltham, Boston, MA, USA) before cDNA preparation. DNase treatment was performed using <1 μg RNA/TNA and incubated at 37 °C for 30 min. The enzymatic reaction was stopped with 0.5M EDTA (pH 8.0) and incubated at 65 °C for an additional ten minutes.

Before conducting the PCR assay, aliquots of four commercial cotton field composite DNA samples were treated with exonuclease V. This enzyme cleaves linear double-stranded DNA in both 5′ and 3′ directions, enabling caulimovirus circular episomal DNA detection [30]. Similarly, TNA from the seeds of two varieties, DG3615 (*n* = 10) and ST4595 (*n* = 10), was treated with exonuclease V to detect the presence of episomal forms of DNA. This treatment was crucial to distinguishing the target episomal DNA from integrated EVE sequences in *G. hirsutum* species. In this treatment, DNA < 1 μg was mixed with Exonuclease V (RecBCD) (NEB, Ipswich, MA, USA) along with ATP and buffers provided by the manufacturer and incubated at 37 °C for 30 min for linear DNA digestion. Later, 0.5 M EDTA (pH 8.0) was added and incubated at 37 °C for an additional 30 min to stop the enzymatic reaction. Linearized DNA digestion of the commercial cotton field samples (S2, S3, S5, S6) was confirmed by analyzing in 0.8% horizontal agarose gel electrophoresis along with untreated DNA and visualized using the gel documentation system (Analytik Jena UVP UVsolo Touch, Upland, CA, USA).

### 2.6. Virus Detection

cDNA was synthesized using superscript III (Invitrogen) using reverse primers for CLRDV capsid protein and P0 gene, TSV capsid, and movement protein gene, followed by PCR using the gene-specific primer pairs (Table 1). An end-point PCR assay was performed targeting the movement protein gene using a primer pair caulimo movement protein primer pair (Caulimo MP-F & Caulimo MP-R) (Table 1) on Exonuclease V-treated DNA samples to detect the presence of episomal DNA. A DNase-RT-PCR assay was performed on the cDNA of the same samples to detect the mRNA transcripts of EVEs. All samples (*n* = 100) collected from the experimental plot were screened for CLRDV-targeting partial capsid protein gene primers in RT-qPCR and for caulimovirus movement protein genes in DNase-RT-PCR using the caulimo movement protein primer pair (Table 1).

### 2.7. High-Throughput Sequencing and Analysis

RNA from four commercial field samples (S2: symptomatic; S3: asymptomatic; S4: symptomatic; and S5: symptomatic) was sent to Novogene, Sacramento, CA, USA, for ribodepletion, cDNA library preparation for single-end reads of (1 × 50 bp) non-coding small RNA (sRNA), and paired-end reads (2 × 150 bp) sequencing of long non-coding RNA (lncRNA) using the Illumina platform.

The sRNA sequence analysis was carried out using the CLC Genomics Workbench (V.23.0.4) (Qiagen, Redwood City, CA, USA). Sequence reads were de novo assembled to create contigs using default parameters. These generated contigs were aligned against the suspected viral sequences using NCBI BLAST. A local virus nucleotide database and a phytoplasma nucleotide database were downloaded on 5 July 2023 from the National Center for Biotechnology Information (NCBI) using the Create Database feature of the CLC Genomics Workbench 23. These contigs were further compared for similarity using the BLASTn [65] tool against all sequences in the database with default parameters set in the CLC Genomics Workbench 23. Sequence reads were mapped with the individual reference sequences of the suspected viruses like CLRDV (NC_014545.1), TSV (KP256522.1), eCPRVE (OR269951) and CotV-A (OR184923). Similarly, in lncRNAs, sequences were trimmed for the adapter and low-quality sequence reads and mapped against CLRDV (NC_014545.1), TSV (KP256522.1), CotV-A (OR184923), and eCPRVE (OR269951) sequences.

Near-complete caulimovirid-like consensus sequences obtained from field samples were compared with the available sequences in NCBI [Accessed in April 2024] of CotV-A, eCPRVE, and other members of the family *Caulimoviridae*. Multiple sequence alignments were performed using a maximum likelihood algorithm using multiple sequence alignment software, MEGA 11 [66]. Among the four EVE sequences obtained, sample PP943202 was used as a reference to mine the plant database, Phytozome (https://phytozome-next.jgi.doe.gov/) [Accessed on 25 May 2024] a Plant Comparative Genomics portal of the Department of Energy’s Joint Genome Institute that consists of updated sequenced genomes of cotton species. The query sequence was compared against the available cotton genome database using the BLAST (BLASTN 2.11.0+) option against the following *Gossypium* species: *G. raimondii*; *G. hirsutum*; *G. mustelinum*_v1_1; *G. tomentosum*_v1_1; *G. barbadense*_v1.1; *G. hirsutum*_v2.1; *G. darwinii*_v1.1; *G. hirsutum*UGA230; *G. hirsutum*UA48; *G. hirsutum*CSX8308; *G. hirsutum*; *G. hirsutum* DeltaPearl; *G. hirsutum*FM958; *G. hirsutum* Coker genome. In addition, the query sequence was also compared with *G. stephensii* (AD7) ‘AD701’, a genome sequence available in the Cottongen database (https://www.cottongen.org) [Accessed on 25 May 2024] that was not reported earlier.

## 3. Results

### 3.1. Symptomatology

**Figure 2:**
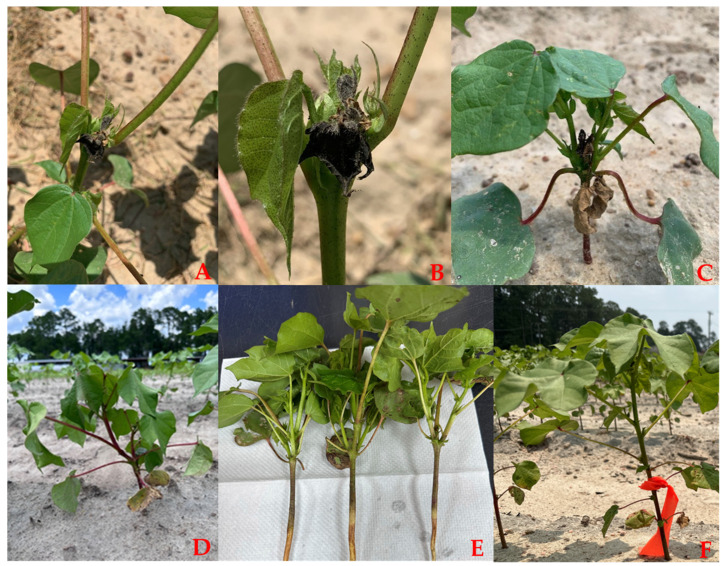
Terminal abortion symptoms in cotton plants; (**A**,**B**) terminal abortion at primary growth stages (2–4 true leaf stage) of cotton seedlings in the field; (**C**) initial stage of terminal abortion resulting in profuse vegetative branching; (**D**) symptomatic plant in the experimental plot in Tift County; (**E**) vegetative branching from Expo-Colquitt and Hopeful-Mitchell counties, GA; (**F**) asymptomatic plant without any vegetative branching. Photo Credit: S.E., P.C. and S.B.

During the growing season of 2023, cotton seedlings (2–4 leaf stage) exhibited terminal abortion (Figure 2A,B), leading to profuse vegetative branching (Figure 2C–E). Samples were collected in two commercial fields and the experimental plot from the plants exhibiting deformed leaf lamina, longer petioles, and stunted plants exhibiting profuse vegetative branching due to terminal abortion (Figure 2D,E) and from the asymptomatic plants devoid of such symptoms (Figure 2F).

### 3.2. Virus Detection

**Figure 3:**
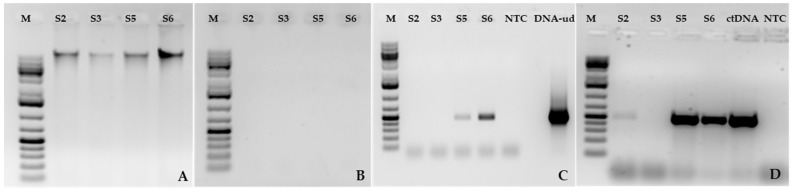
Treated nucleic acid PCR with a caulimo movement protein gene primer pair; (**A**) untreated genomic DNA extracted from pooled field samples; (**B**) exonuclease V digested DNA; (**C**) PCR of Exo V digested DNA; (**D**) DNaseI digested RNA RT-PCR. Lanes are represented as M: Marker; Samples: S2, S3, S5, S6; NTC: No template control; DNA-ud: undigested DNA; ctDNA: cotton DNA.

PCR analysis of commercial field samples (S2: symptomatic; S3: asymptomatic; S5: symptomatic; and S6: asymptomatic) using primer pairs specific for CLRDV and TSV did not amplify any target genes (Figures S1 and S2). The total DNA extracted from samples (Figure 3A) was treated with exonuclease (Figure 3B) and analyzed in horizontal gel electrophoresis. The treated DNA was further subjected to PCR amplification using the caulimovirus movement protein gene, resulting in an amplicon size of approximately 470 bp from Mitchell County (S5: symptomatic; S6: asymptomatic) (Figure 3C) but not in the samples from Colquitt County (S2: symptomatic; S3: asymptomatic). However, a similar amplicon was obtained from both locations except S3 targeting the caulimovirus movement protein gene in DNase-treated RNA RT-PCR (Figure 3C,D), suggesting non-detectable titers of episomal forms along with low RNA transcripts in S2 but none in S3. CLRDV was detected only in two samples (one asymptomatic and the other symptomatic for terminal abortion) among the total collected samples (*n* = 100) tested from the experimental plot. In the same plot, a total of *n* = 96 samples, 93% (37/40) of the symptomatic, 98% (39/40) of the asymptomatic, and 95% (19/20) induced terminal abortion samples were positive for EVE detection. Amplicons were further gel-purified, and the sequence was confirmed through Sanger sequencing, matching 98–100% with eCPRVE (OR269951) and CotV-A (OR184923) partial movement protein gene sequence.

### 3.3. Small and Long Non-Coding RNA Analysis

Four cDNA libraries were prepared and sequenced from four commercial field samples (S2: symptomatic; S3: asymptomatic; S4: symptomatic; and S5: symptomatic). Small RNA reads between 19 and 26 million and long non-coding RNA reads between 57 and 83 million were generated from the samples (Table 2). Mapped small RNA contigs (18–40 bp) were nonspecific and showed no significant matching to the sequences of CLRDV (NC_014545.1), TSV (KP256522.1), eCPRVE (OR269951), and CotV-A (OR184923) (Table 2).

**Figure 4:**
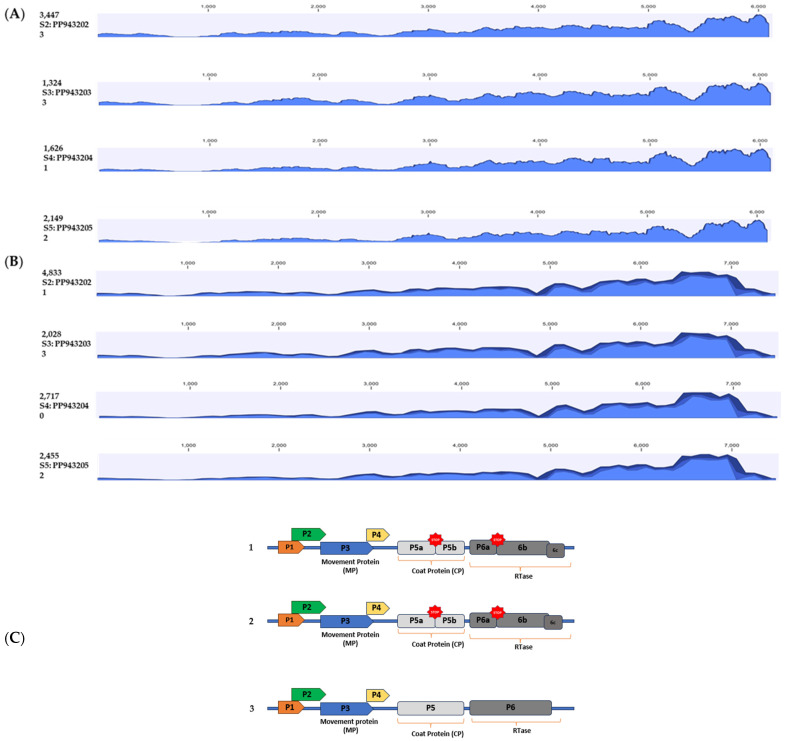
Read coverage map at each genome region, showing the maximum, minimum, and average coverage values with the reference sequence. Scaled genome positions of the virus are represented above the histogram and the Y-axis shows the coverage in number of reads. Within the specified peaks, from top to bottom, the colors represent: the maximum coverage (read counts), the average coverage value, and the minimum coverage value. Read coverage map of field samples EVE-GA’s (S2: PP943202; S3: PP943203; S4: PP943204; and S5: PP943205) with reference sequences (**A**) endogenous cotton pararetroviral elements (eCPRVE; OR269951) and (**B**) cotton virus A (CotV-A; OR184923). Schematic of genome organization of EVE sequences. (**C**) genome organization of endogenous viral elements, GA (1) compared to a putative eCPRVE (2) and CotV-A (3). Stop codons of the open reading frames (ORFs) coding for putative viral coat protein and reverse transcriptases are shown in red spots.

When the paired sequence reads (2 × 150 bp) of long non-coding RNA (lncRNA) of samples (S2: PP943202; S3: PP943203; S4: PP943204; and S5: PP943205) were mapped with near-complete sequences of CotV-A (OR184923) and eCPRVE (OR269951), we obtained 99–100% read coverage (Table 2A). Read maps were graphically visualized by creating read tracks showing maximum, minimum, and average coverage values (Figure 4A,B). Among the 80 million clean reads of S2–S5, 0.02–0.06% were matched with eCPRVE (OR269951) and 0.04–0.09% with CotV-A (OR184923) sequences (Table 2A). However, the lncRNA sequences did not match the reference sequences of CLRDV (NC_014545.1) and TSV (KP256522.1) (Table 2A). The genome organization was represented after comparing the EVE-Georgia (EVE-GA) contigs (PP943202, PP943203, PP943204, and PP943205) with eCPRVE (OR269951) and CotV-A (OR184923). Even though near-complete EVE-GA sequences are 99–100% identical with both eCPRVE and CotV-A (Table 2B), ORF-wise EVE-GA sequences are more similar (99–100%) to the eCPRVE sequence with interruptions due to stopping codons in open reading frames (ORFs) that code for putative viral coat proteins and reverse transcriptase (Figure 4C).

### 3.4. Validation of HTS Results

In commercial field samples (*n* = 54) testing, the caulimovirus movement protein gene was detected in both symptomatic and asymptomatic plants from both locations. The presence of the movement protein gene was confirmed in 13 of the 24 symptomatic samples in Colquitt and 15 of the 22 symptomatic samples from Mitchell County. In contrast, it was detected in all the asymptomatic plants in both locations. (Table 3).

### 3.5. BLAST, Phylogenetic Analysis, and In Silico Mining

**Figure 5:**
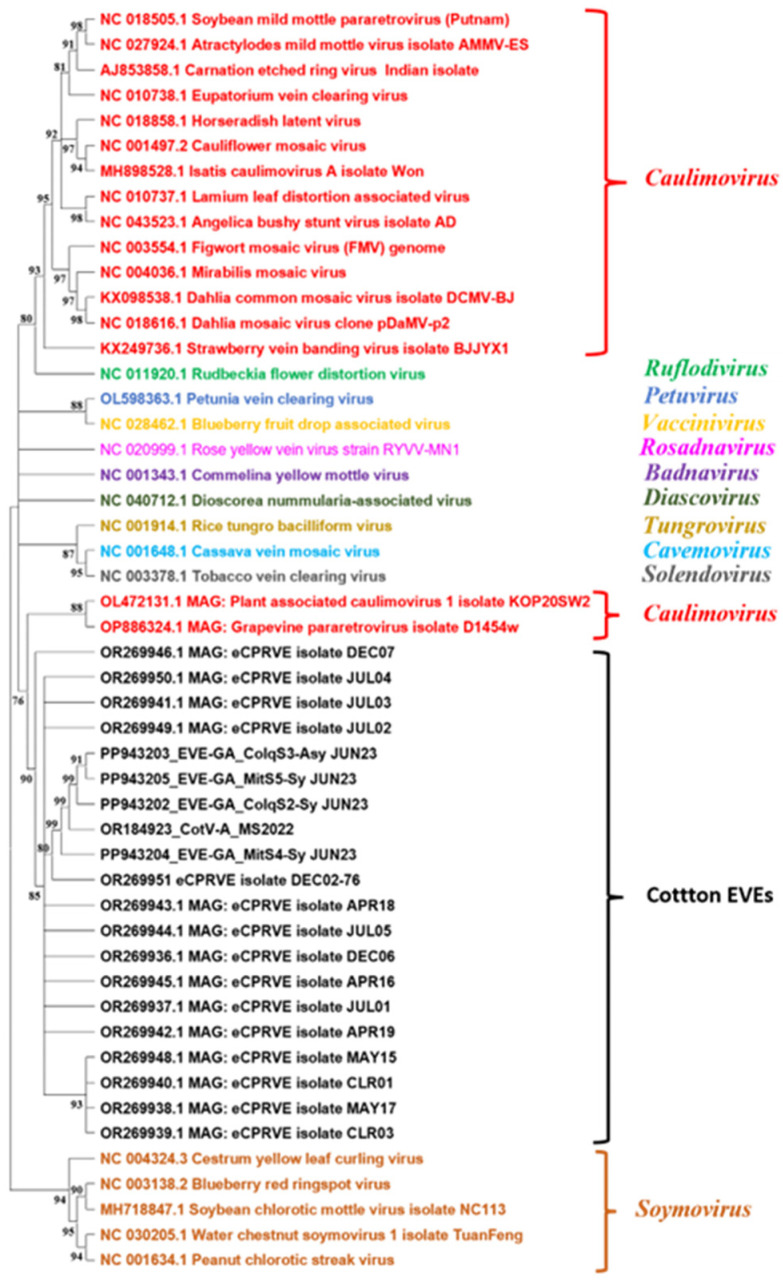
Phylogenetic tree comparing near-complete sequences of endogenous viral elements (EVEs) obtained from field samples (S2: PP943202; S3: PP943203; S4: PP943204; and S5: PP943205) of GA in comparison to near-complete sequences of cotton virus A isolate, endogenous cotton pararetroviral elements (eCPRVE) DEC02-76 isolate, and various genera in the family *Caulimoviridae*. Color code represents similar genera.

Consensus sequences from the lncRNA sequence of the symptomatic samples collected from growers’ fields did not exhibit any matches with CLRDV (NC_014545.1) and Ilarviruses, TSV (KP256522.1), when analyzed with the CLC workbench. The sequence matched (98%) with CotV-A and eCPRVE sequences when mapped against the sequences available with NCBI GenBank. In the phylogenetic analysis, the nucleotide sequences of the near-complete sequence from commercial field isolates from GA were 90–98% identical with eCPRVE sequences (OR269936 to OR269951) and the DNA virus CotV-A (OR184923) reported earlier from Mississippi, USA. Further, the EVE sequences from GA are 88% identical to those of the caulimovirus members like plant-associated caulimovirus (OL472131) and grapevine para retrovirus (OP886324). These sequences form a distinct clade from the members of the family *Caulimoviridae* (Figure 5). In our data mining using EVE query (PP943202) sequences obtained from HTS, we found near-identical integrated sequences in tetraploid species of *G.hirsutum* cultivars with triplets of high-scoring segment pair (HSPs) of EVEs in A04 chromosome (+/−) with ~7 kb, ~6 kb, and 394 bp lengths showing 97–100% identity (Supplementary Table S1). In addition, we also observed the integrated near-identical EVE sequences in other *G. hirsutum* chromosomes with various matching lengths and percentage identity (Chromosome-D03 (+/+): ~4 kb with 84% identity, A05 (+/+): ~3 kb with 73% identity, D07 (+/−): 1059 bp with 80% identity, A13 (+/−): 1254 bp with 76% identity).

Other than *G. hirsutum* species, we also observed EVE sequences in *G. stephensii* chromosomes A04 with two HSPs in + strands, one with ~7 kb, and the other ~6.2 kb with 99% identity, D03 (+/+): ~3.6 kb with 84% identity, D07 (+/+): ~1 kb with 80% identity, *G. barbadense*, D04 chromosome (+/+): ~1 kb, *G. tomentosum* in A03 (+/+) ~5.3 kb with 79% identity, D04, A13 chromosomes, and *G. mustelinum* in D07 (+/+): ~5.1 kb with 70% identity, and D05, A07 chromosome consisting various lengths of EVE sequence. In the in silico mining of the cotton genome database, the sequences obtained via HTS data appear to be from a negative-sense copy of EVEs, as described in Aboughanem-Sabanadzovic et al. [25].

### 3.6. Cotton Yield Components

In DG3615, the yield components, including seedcotton yield and boll density, demonstrated no significant difference between symptomatic and asymptomatic plants (Table 4). Similarly, in DGH959, seedcotton yield and boll density demonstrated no significant difference when compared between different treatments (symptomatic, asymptomatic, and induced terminal abortion) (Table 4). These results suggest that the terminal abortion symptom observed in the growing season of 2023 did not result in a yield reduction with respect to the varieties tested.

### 3.7. Seed and Seedling Assay

The Caulimovirus movement protein gene was detected in different tissues of the DG3615, DGH959, DP1646, and ST4595 varieties in both germinated seed and seedling stages. In seed and seedling testing, 95% of seeds (38/40) and 75% (30/40) of seedlings were positive for EVE detection (Table 3). We detected the caulimovirus movement protein gene in a higher percentage, 85% (34/40) in plumule and epicotyl, followed by 65% (26/40) in seed coat, and 53% (21/40) in hypocotyl and root tissue. However, in the seedling analysis, cotyledon leaves were 65% (26/40), stem tissues were 63% (25/40), and root tissues were 58% (23/40) positive for caulimovirus movement protein gene detection. Among the assessed varieties, DG3615 and DGH959 had comparatively higher percentages (66%) of positives for EVE detection in both seeds and seedlings compared to ST4595 and DP1646 (31%). In the exonuclease V-treated samples of DG3615 and ST4595 (*n* = 20), 100% (10/10) of samples were positive for the caulimovirus movement protein gene detection in cotyledon leaves.

## 4. Discussion

In this study, we evaluated the terminal abortion-symptomatic plants that appeared sporadically in the growing season of 2023 in Tift, Mitchell, and Colquitt County, GA. Concurrently, the identification of EVEs in cotton [25,56] solicited the question of whether these elements could play a role in terminal abortion leading to profuse vegetative branching. To enhance our understanding of EVE presence in Georgia-grown cotton, we investigated and detected near-complete sequences (~7.4 kb) in both symptomatic (terminal abortion) and asymptomatic samples. Typically, the terminal abortion of cotton arises from abiotic factors such as wind and hail damage. On occasion, it can also be triggered by biotic elements, including insect feeding, such as tarnished plant bugs commonly found in weed hosts like Palmer amaranth (*Amaranthus palmeri* S. Watson) [67]. Additionally, sucking pests like thrips (*Frankliniella fusca* Hinds) can induce terminal abortion by feeding on slow-growing cotton seedlings at cold temperatures [68,69]. However, our observations in the fields and thrips infestation predictors indicated that the population of thrips was significantly low in the fields during the early weeks of June 2023 [68,70], potentially due to recurrent rainfalls and routine prophylactic management practices [71,72] implemented at the onset of each crop season.

Based on previous studies, there are instances where replication-competent EVEs are induced by various factors such as genome hybridization, tissue culture, abiotic stress, and wounding. This occurrence has been discovered in hosts like banana (endogenous banana streak viruses, eBSVs) [35,36], tobacco (endogenous tobacco vein-clearing virus, eTVCV) [73] and petunia sps. (endogenous petunia vein-clearing virus, ePVCV) [74,75,76]. In some cases, EVEs are incapable of autonomous replication due to deficiencies in structural domains, but sometimes their replication is supported by co-infecting viruses from the same family [77]. Thus, the samples were tested for the presence of viruses, including CLRDV, TSV, or EVEs (CotV-A and eCPRVE), which were suspected to be the potential causal agents of terminal abortion. The absence of suspected viruses like CLRDV and TSV in the RT-PCR assays of samples collected from commercial cotton fields suggests their non-involvement during terminal abortion. The absence of CLRDV in the UGA-Bowen farm experimental field, except for one symptomatic (terminal abortion) and one asymptomatic sample, indicates that CLRDV was not prevalent and unlikely to be a causal agent for terminal abortion. Further, in the Bowen farm experimental plot, the EVE detection rate was over 90% across the treatments (symptomatic, asymptomatic, and induced terminal abortion), despite showing differences among them. This supports the hypothesis that EVEs are unlikely to be the causal agent for terminal abortion.

To comprehend the functional status of recently identified cotton EVEs, we analyzed their episomal forms and mRNA transcripts using the protocol of caulimo movement protein gene primers for CotV-A, as described by Ortiz et al. [56]. Our analysis revealed the presence of EVEs in the episomal forms in Mitchell County samples but not in Colquitt County. Messenger RNA transcripts of the caulimo movement protein gene were detected in both symptomatic and asymptomatic samples from Mitchell County but only in symptomatic samples from Colquitt County, and non-detectable in the asymptomatic sample from Colquitt County. These results indicate that the formation of episomal DNA from host-integrated sequences may be inconsistent and/or likely a redundant expression and cannot be directly linked to the cause of terminal abortion. As investigated by Squires et al. [78]**,** the episomal DNA expression of the cauliflower mosaic virus (CaMV) infectious clone in Arabidopsis was not temperature-dependent. Similarly, our results corroborate the detection of EVEs’ episomal DNA in both symptomatic and asymptomatic field samples exposed to environmental stress (temperature differences) and seeds independent of environmental stress.

In HTS analysis of sRNA and lncRNA sequences, CLRDV and TSV were not detected. However, near-complete, caulimovirid-like sequences were detected for eCPRVE and CotV-A in lncRNAs but not in sRNAs. Commonly, lncRNAs are moderately abundant fractions of eukaryotic transcriptomes (>200 nt) that are lacking coding capacity but are involved in plant gene regulation, and some act as positive or negative regulators of plant immunity [79,80]. In comparison, sRNAs are microRNAs (18–40 nt), usually non-coding, and involved in antiviral immunity by guiding argonaut proteins to target viral RNA cleavage [58]. HTS assays are widely used for the comprehensive assessment of pathogen profiles. They play a crucial role in the discovery of emerging, reemerging, and mixed viral infections in both cultivated crops and wild plant species [81,82]. Many plants respond to exogenous virus infections via transcriptional (TGS) and post-transcriptional gene silencing (PTGS), with PTGS occurring in the cytoplasm and targeting dsRNA intermediates. Although DNA viruses replicate in the nucleus, mRNAs are transported to the cytoplasm for translation or for reverse transcription (pararetorviruses), which makes them potential PTGS targets [83]. Despite this, the absence of such sRNAs of EVE sequences related to eCPRVEs, CotV-A, or any persistent viruses [62,63] in high-throughput-based sRNA sequences disproves their involvement in terminal abortion. The nonexistence of viral sRNA sequences in the symptomatic and asymptomatic samples, even in the more sensitive HTS, confirms the absence of an active host defense response against the viruses. This inactivity could be attributed to EVE sequences losing infectivity due to various aspects such as insertions and deletions (indels), mutations, and fragmentation during host genome replication [25].

The detection of near full-length EVE sequences in the cotton samples (symptomatic and asymptomatic) collected from growers’ fields in Colquitt and Mitchell counties in GA implies its presence in the varieties of *Gossypium hirsutum* L. genetic background [25]. Moreover, the sequences we obtained consisted of multiple ORFs similar to those submitted by Aboughanem-Sabanadzovic et al. [25], which are capable of encoding various proteins. However, the sequences from GA had stop codons in the open reading frames 5 and 6 (ORFs) coding for putative viral coat protein and reverse transcriptases similar to eCPRVEs as described in Aboughanem-Sabanadzovic et al. [25], making them non-functional. Usually, in caulimovirus, mRNA is polycistronic and translated via ribosomal reinitiation [84]. Interestingly, no such interruptions due to stop codons were observed in the CotV-A sequence, which requires further evaluation to confirm the functional status of these ORFs [56].

**Figure 6:**
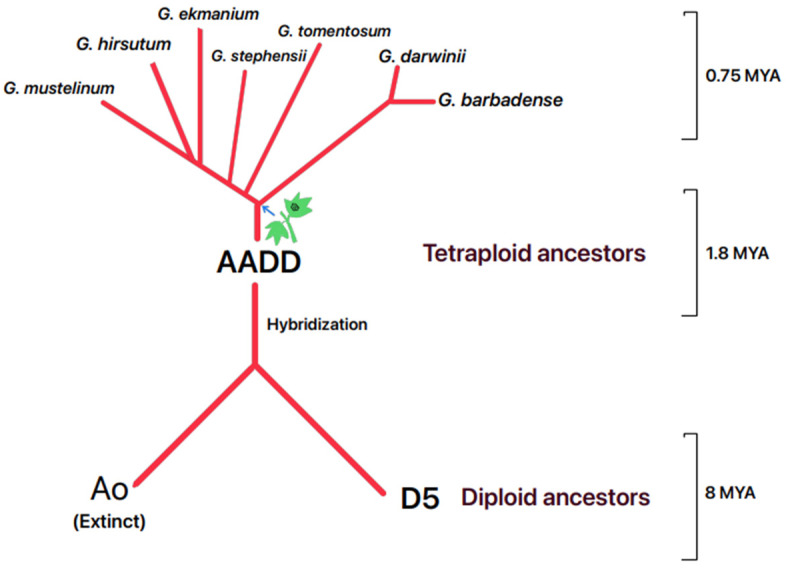
Graphic representation of the origin of tetraploid *Gossypium* spp. from their diploid ancestors indicated with timeline and possible virus integration event represented using a green cotton plant and a blue arrow.

In silico mining of the EVE sequences in the cotton genome database strongly manifests the presence of endogenous viral sequences in various species of *Gossypium* L., which is not limited to the hirsutum species prominent in North and South Americas but also to the species present in Australia (*G.hirsutum* CSX8308). Caulimovirus-like near-complete EVE sequences were also found in *G. stephensii* A04 and D03 chromosomes, showing similar matches to other hirsutum species. This supports the hypothesis of integration predating the speciation events of *G. hirsutum*, estimated at 0.75 mya, as speculated in Aboughanem-Sabanadzovic et al. [25]. However, EVE integrations of approximately ~5 kb with 79% identity were found in Chr A03 in *G. tomentosum* and about ~5 kb with 70% identity in Chr D07 in *G. mustelinum*. These integrations were also present in two other species of tetraploids (*G.barbadense* and *G. darwinii*) in very minimal-length ~1 kb sequences with 70% identity (Table S1). This shows a high degree of sequence degradation, raising a query about whether the integration event was much earlier (1.80 mya) (Figure 6) than the hirsutum speciation event in the tetraploid “AADD” ancestors or a recent (0.75 mya) multiple independent integrations that needs further investigation. Identifying active mRNA transcripts of ORF3 (movement protein) [25] in EVEs only partially evaluates the entire polycistronic mRNA. A deeper insight into the genomic annotations and functions of other ORFs can significantly enhance our understanding of EVEs in cotton. However, additional investigation is essential to ascertain if episomal DNAs are involved in virion formation and infectivity. Although eCPRVE sequences were discovered in CLRDV-infected cotton plants [25], any correlation (synergistic or antagonistic) between these two viruses has not been established yet. Such clarification on their synergism or antagonism is vital in understanding their role in infection and symptom development within the host plant.

In a study of *Dahlia variabilis* endogenous pararetrovirus sequence (DvEPRS), integrated into the host dahlia (*D. variabilis*) genome, it was detected in various tissues, including leaves, roots, seeds, flower petals, and pollen, and was capable of 100% seed transmission [27,77,85]. Similarly, our results indicate the presence of EVEs in seeds and seedlings, expressed as episomal forms and mRNA transcripts, although their presence in other tissues was not tested. However, the transmission of DvEPRS by mechanical inoculation and through aphids (*Myzus persicae*) was unsuccessful [77]. Further research is imperative to understand such prospects with EVEs in cotton.

To address the concern about the emerging issue of terminal abortion resulting in profuse vegetative branching, yield impact was assessed in two varieties (DG3615 and DGH959) at an experimental plot at the UGA Bowen research farm in Tifton, GA. The results showed no significant difference in the yield response variables: seedcotton yield (DG3615: *p* = 0.4139, DGH959: *p* = 0.8866) and boll density (DG3615: *p* = 0.4933, DGH959: *p* = 0.7028) between the treatments of two varieties. These findings imply that the terminal abortion leading to profuse vegetative branching observed in the growing season of 2023 did not adversely affect the yield in the varieties tested, which further supports the speculation that terminal abortions may not be concerning at present and the plants are likely to recover to sustain yields. Despite the inconclusive findings of the exact causal agent for terminal abortion, it is worthwhile to explore the cotton genotype response to climate change and increasing temperatures. Consequently, our study provides valuable insights for cotton growers and researchers into the significance of caulimovirid-like EVEs in the cotton genome, paving the way for future research on EVEs to assess their activity and involvement in host interactions. In some cases, putative EVEs may confer host resistance to associated viral infections [86,87,88,89]. The discovery of EVE sequences in lncRNAs, but not in small RNAs, prompts the intriguing question of whether they have a role in conferring host immunity, which will be an interesting aspect that needs to be substantiated in future research.

## 5. Conclusions

During the 2023 growing season in Georgia, USA, the intermittent appearance of terminal abortion in young cotton plants with no apparent cause raised concerns among industry and academic scientists alike. Therefore, samples were evaluated for multiple aspects to address these concerns. The impact of terminal abortion on cotton yield was mainly assessed, and there was no significant difference between symptomatic and asymptomatic plants. The association between tarnished plant bugs and thrips was also non-significant at the time of symptom appearance. This study further evaluated the presence of EVEs in the cotton genome, finding no correlation between their presence and the occurrence of terminal abortion. Further research and evaluation of cotton EVEs is needed to understand their true functionality and role in pathogenicity or immunity.

## Figures and Tables

**Table 1:** Oligo primer and the targeted virus genes used in this study.

S. No	Oligo	Virus	Gene Targeted	Primer Sequence	References
1	Caulimo MP-F	EVEs	Movement protein	GGACGACTCGAAGGAAACTTAGG	[56]
2	Caulimo MP-R	EVEs	Movement protein	ACTAGAAGGGTGCTCTACTGGTA	[56]
3	SB26-F	CLRDV	Partial Capsid protein	CTCAATGGTCTTATTGGAGTTCA	[47]
4	SB26-R	CLRDV	Partial Capsid protein	TTCCTCCCATTCTTGGTGATTCC	[47]
5	SB11-F	CLRDV	Capsid protein	AGGTTTTCTGGTAGCAGTACCAATATCAACGTTA	[64]
6	SB11-R	CLRDV	Capsid protein	TATCTTGCATTGTGGATTTCCCTCATAA	[64]
7	SB 28-F	CLRDV	P0 protein	CACTTGAGACATAACTCGCTT	[64]
8	SB 28-R	CLRDV	P0 protein	GCGGTGAGGAGACCATACTCA	[64]
9	SB162F	TSV	Capsid protein	TCAGCCTGACTGTTGGGTTGT	[54]
10	SB162R	TSV	Capsid protein	AGCTATGCATGTTGTTCATAGG	[54]
11	SB164F	TSV	Movement protein	ACGATTTCCAACTTTGAATTCCTACAA	[54]
12	SB164R	TSV	Movement protein	ATCTATCTCTAGAATTCATCAACTTAATACT	[54]

**Table 2:** Long non-coding and small RNAs read coverage, matching, and percent nucleotide identity with different virus sequences suspected in the occurrence of terminal abortion.

**(A)**												
**Sample ID**	**Total lncRNA Reads**	**Reads Match to CotV-A**	**Consensus seq**	**Coverage% ** **with CotV-A**	**Nucleotide % Identity**	**Reads Match to eCPRVE**	**Coverage % with eCPRVE**	**Nucleotide % Identity**	**Reads Matching CLRDV**	**Coverage % with CLRDV**	**Reads Matching with TSV**	**Coverage % with TSV**
S2	78,686,152	73,027 (0.09)	7484	100	99.55	47,584 (0.06)	100	99.84	0	-	0	-
S3	57,242,296	29,794 (0.05)	7481	99.99	99.45	19,663 (0.03)	100	99.72	0	-	0	-
S4	83,642,364	33,534 (0.04)	7481	99.99	99.44	20,928 (0.02)	100	99.72	0	-	0	-
S5	83,991,624	43,588 (0.05)	7482	100	99.49	26,587 (0.03)	100	99.77	0	-	0	-
**(B)**												
**Sample ** **ID**	**Total sRNA reads**	**% sRNA reads match with** **CotV-A**		**Coverage % with CotV-A**	**% sRNA reads** **match with ** **eCPRVE**		**Coverage % with eCPRVE**		**% sRNA reads ** **match with** **CLRDV**	**Coverage % ** **with CLRDV**	**% sRNA reads match** **with TSV**	**Coverage % with TSV**
**S2**	22,664,462	1462 (0.006)		-	1300 (0.005)		-		539 (0.002)	-	330 (0.001)	-
**S3**	19,209,722	985 (0.005)		-	873 (0.004)		-		395 (0.002)	-	237 (0.001)	-
**S4**	22,718,020	2138 (0.009)		-	1833 (0.008)		-		944 (0.004)	-	663 (0.002)	-
**S5**	26,555,305	2169 (0.008)		-	1904 (0.007)		-		893 (0.003)	-	643 (0.002)	-

The table above represents the coverage of Table 2A long non-coding RNAs with eCPRVE (OR269951), CotV-A (OR184923), CLRDV (NC_014545.1), and TSV (KP256522.1) sequences; Table 2B small RNA sequences of field samples with eCPRVE (OR269951), CotV-A (OR184923), CLRDV (NC_014545.1) and TSV (KP256522.1). Abbreviation used: eCPRVE: endogenous cotton pararetroviral elements; CotV-A: cotton virus A; CLRDV: cotton leafroll dwarf virus; TSV: tobacco streak virus.

**Table 3:** Endogenous viral elements detection in cotton samples from field and greenhouse conditions.

Cotton Sample-Type	Number of Samples Tested (*n*)	^a^ Number of Samples (*n*) Positive in PCR/RT-PCR/qPCR		
		EVEs	CLRDV	TSV
**Commercial field samples**				
Symptomatic (L1&2)	46	32 (70%)	ND	ND
Asymptomatic (L1&2)	8	8 (100%)	ND	ND
**Experimental plot**				
Symptomatic	40	37 (93%)	1 (2.5%)	NT
Asymptomatic	40	39 (98%)	1 (2.5%)	NT
Induced	20	19 (95%)	ND	
**Seed assessment (Greenhouse conditions)**				
Seeds	40	38 (95%)	NT	NT
Seedlings	40	30 (75%)	NT	NT

^a^ Sample percentage is rounded off to the nearest decimal; Acronyms used are ND-not detected and NT-not tested. Abbreviation used: EVEs: endogenous viral elements; CLRDV: cotton leafroll dwarf virus; TSV: tobacco streak virus.

**Table 4:** Yield response of two Dyna-Gro varieties: DG3615 and DGH959.

Variety	Treatment	Seedcotton Yield (g plant^−1^)	Boll Density plant^−1^
DG3615	Asymptomatic	51.97 a	9.85 a
	Symptomatic	43.53 a	8.5 a
	*p* value	**0.4139**	**0.4933**
DGH959	Asymptomatic	60.57 a	12.3 a
	Symptomatic	57.36 a	11.3 a
	Induced terminal abortion	56.55 a	10.95 a
	*p* value	**0.8866**	**0.7028**

Alphabet (a) represents the compact letter display showing statistical significance.

## Data Availability

Data are contained within the article.

## Supplementary Materials

The following supporting information can be downloaded at: https://www.mdpi.com/article/10.3390/v16071111/s1. Table S1: In silico mining of integrated caulimovirid-like EVE sequences in the various Gossypium sps. using cotton databases; Figure S1: Detection of tobacco steak virus (TSV) using RT-PCR, (A) coat protein gene using primer pair SB162 F/R and (B) movement protein gene using primer pair SB164 F/R; M- Marker, Samples: S2, S3, S5, S6, NTC-No template control; Figure S2: Detection of cotton leafroll dwarf virus (CLRDV) using RT-PCR, (A) P0 protein gene using primer pair SB28 F/R; (B) Coat protein gene detection using primer pair SB11F/R; Lanes were marked as: M-Marker; Samples: S2, S3, S5, S6; HC-Healthy control; PC1 and PC2-plant positive control; EC-P0 amplicons eluted from prior PCR used as positive control for P0 gene, NTC-No template control.

## References

[1] Bellas C., Hackl T., Plakolb M.-S., Koslová A., Fischer M.G., Sommaruga R. (2023). Large-Scale Invasion of Unicellular Eukaryotic Genomes by Integrating DNA Viruses. Proc. Natl. Acad. Sci. USA.

[2] Veglia A.J., Bistolas K.S.I., Voolstra C.R., Hume B.C.C., Ruscheweyh H.-J., Planes S., Allemand D., Boissin E., Wincker P., Poulain J. (2023). Endogenous Viral Elements Reveal Associations between a Non-Retroviral RNA Virus and Symbiotic Dinoflagellate Genomes. Commun. Biol..

[3] Koslová A., Hackl T., Bade F., Sanchez Kasikovic A., Barenhoff K., Schimm F., Mersdorf U., Fischer M.G. (2024). Endogenous Virophages are Active and Mitigate Giant Virus Infection in the Marine Protist *Cafeteria burkhardae*. Proc. Natl. Acad. Sci. USA.

[4] Zhao H., Zhang R., Wu J., Meng L., Okazaki Y., Hikida H., Ogata H. (2023). A 1.5-Mb Continuous Endogenous Viral Region in the Arbuscular Mycorrhizal Fungus *Rhizophagus irregularis*. Virus. Evol..

[5] Flynn P.J., Moreau C.S. (2019). Assessing the Diversity of Endogenous Viruses throughout Ant Genomes. Front. Microbiol..

[6] Liu S., Coates B.S., Bonning B.C. (2020). Endogenous Viral Elements Integrated into the Genome of the Soybean Aphid, *Aphis glycines*. Insect Biochem. Mol. Biol..

[7] Suzuki Y., Baidaliuk A., Miesen P., Frangeul L., Crist A.B., Merkling S.H., Fontaine A., Lequime S., Moltini-Conclois I., Blanc H. (2020). Non-Retroviral Endogenous Viral Element Limits Cognate Virus Replication in *Aedes aegypti* Ovaries. Curr. Biol..

[8] Huang H.-J., Li Y.-Y., Ye Z.-X., Li L.-L., Hu Q.-L., He Y.-J., Qi Y.-H., Zhang Y., Li T., Lu G. (2023). Co-Option of a Non-Retroviral Endogenous Viral Element in Planthoppers. Nat. Commun..

[9] Naville M., Volff J.N. (2016). Endogenous Retroviruses in Fish Genomes: From Relics of Past Infections to Evolutionary Innovations?. Front. Microbiol..

[10] Cui J., Zhao W., Huang Z., Jarvis E.D., Gilbert M.T.P., Walker P.J., Holmes E.C., Zhang G. (2014). Low Frequency of Paleoviral Infiltration Across the Avian Phylogeny. Genome Biol..

[11] Mason A.S., Lund A.R., Hocking P.M., Fulton J.E., Burt D.W. (2020). Identification and Characterisation of Endogenous Avian Leukosis Virus Subgroup E (ALVE) Insertions in Chicken Whole Genome Sequencing Data. Mob. DNA.

[12] Bejarano E.R., Khashoggi A., Witty M., Lichtenstein C. (1996). Integration of Multiple Repeats of Geminiviral DNA into the Nuclear Genome of Tobacco during Evolution. Proc. Natl. Acad. Sci. USA.

[13] Belyi V.A., Levine A.J., Skalka A.M. (2010). Sequences from Ancestral Single-Stranded DNA Viruses in Vertebrate Genomes: The Parvoviridae and Circoviridae are more than 40 to 50 Million Years Old. J. Virol..

[14] Katzourakis A., Gifford R.J. (2010). Endogenous Viral Elements in Animal Genomes. PLoS Genet..

[15] Weiss R. (1967). Spontaneous Virus Production from “Non-Virus Producing” Rous Sarcoma Cells. Virol. J..

[16] Stein R.A., DePaola R.V. (2023). Human Endogenous Retroviruses: Our Genomic Fossils and Companions. Physiol. Genom..

[17] Katoh H., Honda T. (2023). Roles of Human Endogenous Retroviruses and Endogenous Virus-like Elements in Cancer Development and Innate Immunity. Biomolecules.

[18] Skirmuntt E.C., Escalera-Zamudio M., Teeling E.C., Smith A., Katzourakis A. (2020). The Potential Role of Endogenous Viral Elements in the Evolution of Bats as Reservoirs for Zoonotic Viruses. Annu. Rev. Virol..

[19] Weiss R.A. (2006). The Discovery of Endogenous Retroviruses. Retrovirology.

[20] Andrake M.D., Skalka A.M. (2015). Retroviral Integrase: Then and Now. Annu. Rev. Virol..

[21] Ashby M.K., Warry A., Bejarano E.R., Khashoggi A., Burrell M., Lichtenstein C.P. (1997). Analysis of Multiple Copies of Geminiviral DNA in the Genome of Four Closely Related Nicotiana Species Suggest a Unique Integration Event. Plant Mol. Biol..

[22] Jakowitsch J., Mette M.F., van der Winden J., Matzke M.A., Matzke A.J.M. (1999). Integrated Pararetroviral Sequences Define a Unique Class of Dispersed Repetitive DNA in Plants. Proc. Natl. Acad. Sci. USA.

[23] Diop S.I., Geering A.D.W., Alfama-Depauw F., Loaec M., Teycheney P.-Y., Maumus F. (2018). Tracheophyte Genomes Keep Track of the Deep Evolution of The Caulimoviridae. Sci. Rep..

[24] Geering A.D.W., Maumus F., Copetti D., Choisne N., Zwickl D.J., Zytnicki M., McTaggart A.R., Scalabrin S., Vezzulli S., Wing R.A. (2014). Endogenous Florendoviruses are Major Components of Plant Genomes and Hallmarks of Virus Evolution. Nat. Commun..

[25] Aboughanem-Sabanadzovic N., Allen T.W., Frelichowski J., Scheffler J., Sabanadzovic S. (2023). Discovery and Analyses of Caulimovirid-like Sequences in Upland Cotton (*Gossypium hirsutum*). Viruses.

[26] Holmes E.C. (2011). The Evolution of Endogenous Viral Elements. Cell Host Microbe.

[27] Pahalawatta V., Druffel K., Pappu H. (2008). A New and Distinct Species in the Genus Caulimovirus Exists as an Endogenous Plant Pararetroviral Sequence in its Host, *Dahlia variabilis*. Virology.

[28] Gayral P., Iskra-Caruana M.-L. (2009). Phylogeny of Banana Streak Virus Reveals Recent and Repetitive Endogenization in the Genome of Its Banana Host (*Musa* sp.). J. Mol. Evol..

[29] Krupovic M., Blomberg J., Coffin J.M., Dasgupta I., Fan H., Geering A.D., Gifford R., Harrach B., Hull R., Johnson W. (2018). Ortervirales: New Virus Order Unifying Five Families of Reverse-Transcribing Viruses. Virol. J..

[30] Teycheney P.Y., Geering A.D.W., Dasgupta I., Hull R., Kreuze J.F., Lockhart B., Muller E., Olszewski N., Pappu H., Pooggin M.M. (2020). ICTV Virus Taxonomy Profile: Caulimoviridae. J. Gen. Virol..

[31] Hull R. (2014). Profiles of Families and Genera of Plant Viruses. Plant Virology.

[32] Pfeiffer P., Hohn T. (1983). Involvement of Reverse Transcription in the Replication of Cauliflower Mosaic Virus: A Detailed Model and Test of Some Aspects. Cell.

[33] Schoelz J.E., Mahy B.W.J., Van Regenmortel M.H.V. (2008). Caulimoviruses: General Features. Encyclopedia of Virology.

[34] Scholthof H.B., Gowda S., Wu F.C., Shepherd R.J. (1992). The Full-Length Transcript of a Caulimovirus is a Polycistronic mRNA Whose Genes are Trans Activated by the Product of Gene VI. J. Virol..

[35] Ndowora T., Dahal G., LaFleur D., Harper G., Hull R., Olszewski N.E., Lockhart B. (1999). Evidence that Badnavirus Infection in *Musa* Can Originate from Integrated Pararetroviral Sequences. Virology.

[36] Harper G., Osuji J.O., Heslop-Harrison J.S., Hull R. (1999). Integration of Banana Streak Badnavirus into the *Musa* Genome: Molecular and Cytogenetic Evidence. Virology.

[37] Chabannes M., Baurens F.C., Duroy P.O., Bocs S., Vernerey M.S., Rodier-Goud M., Barbe V., Gayral P., Iskra-Caruana M.L. (2013). Three Infectious Viral Species Lying in Wait in the Banana Genome. J. Virol..

[38] Saito N., Chen S., Kitajima K., Zhou Z., Koide Y., Encabo J.R., Diaz M.G.Q., Choi I.R., Koyanagi K.O., Kishima Y. (2023). Phylogenetic Analysis of Endogenous Viral Elements in the Rice Genome Reveals Local Chromosomal Evolution in Oryza AA-Genome Species. Front. Plant Sci..

[39] Chen S., Saito N., Encabo J.R., Yamada K., Choi I.R., Kishima Y. (2018). Ancient Endogenous Pararetroviruses in Oryza Genomes Provide Insights into the Heterogeneity of Viral Gene Macroevolution. Genome Biol. Evol..

[40] Muller E., Ullah I., Dunwell J.M., Daymond A.J., Richardson M., Allainguillaume J., Wetten A. (2021). Identification and Distribution of Novel Badnaviral Sequences Integrated in the Genome of Cacao (*Theobroma cacao*). Sci. Rep..

[41] Gong Z., Han G.Z. (2018). Euphyllophyte Paleoviruses Illuminate Hidden Diversity and Macroevolutionary Mode of Caulimoviridae. J. Virol..

[42] Yu H., Wang X., Lu Z., Xu Y., Deng X., Xu Q. (2019). Endogenous Pararetrovirus Sequences are Widely Present in Citrinae Genomes. Virus Res..

[43] Schmidt N., Seibt K.M., Weber B., Schwarzacher T., Schmidt T., Heitkam T. (2021). Broken, Silent, and in Hiding: Tamed Endogenous Pararetroviruses Escape Elimination from the Genome of Sugar Beet (*Beta vulgaris*). Ann. Bot..

[44] de Tomás C., Vicient C.M. (2022). Genome-Wide Identification of Reverse Transcriptase Domains of Recently Inserted Endogenous Plant Pararetrovirus (Caulimoviridae). Front. Plant Sci..

[45] Mahmood M.A., Ahmed N., Hussain A., Naqvi R.Z., Amin I., Mansoor S. (2024). Dominance of Cotton leaf curl Multan virus-Rajasthan Strain Associated with Third Epidemic of Cotton Leaf Curl Disease in Pakistan. Sci. Rep..

[46] Rageshwari S., Malathi V.G., Renukadevi P., Nakkeeran S. (2023). Molecular Studies on Tobacco Streak Virus (TSV) Infecting Cotton in Tamil Nadu, India. 3 Biotech.

[47] Edula S.R., Bag S., Milner H., Kumar M., Suassuna N.D., Chee P.W., Kemerait R.C., Hand L.C., Snider J.L., Srinivasan R. (2023). Cotton Leafroll Dwarf Disease: An Enigmatic Viral Disease in Cotton. Mol. Plant Pathol..

[48] Culbreath A.K., Todd J.W., Demski J.W. (1992). Productivity of Florunner Peanut Infected with Tomato Spotted Wilt Virus. Peanut Sci..

[49] Gitaitis R.D., Dowler C.C., Chalfant R.B. (1998). Epidemiology of Tomato Spotted Wilt in Pepper and Tomato in Southern Georgia. Plant Dis..

[50] McDaniel L.L., Raid R.N., Elliott C.L., Tsai J.H., Nagata R.T. (1992). Purification and Serological Characterization of a Tobacco Streak Virus Isolate Infecting Field-Grown Escarole and Lettuce. Plant Dis..

[51] Sharman M., Persley D.M., Thomas J.E. (2009). Distribution in Australia and Seed Transmission of Tobacco streak virus in *Parthenium hysterophorus*. Plant Dis..

[52] Hosseini S., Habibi M.K., Mosahebi G., Motamedi M., Winter S. (2012). First Report on The Occurrence of Tobacco Streak Virus in Sunflower in Iran. Plant Pathol. J..

[53] Daliyamol, Jailani A.A.K., Vemana K., Roy A., Krishnareddy M., Kobayashi K., Mandal B. (2019). Complete Genome Sequence and Phylogenetic Relationships of Tobacco Streak Virus Causing Groundnut Stem Necrosis Disease in India. Virusdisease.

[54] Bag S., Tabassum A., Brock J., Dutta B. (2019). First Report of Tobacco Streak Virus Infecting Summer Squash in Georgia, U.S.A. Plant Dis..

[55] Zambrana-Echevarría C., Roth M.G., Dasgupta R., German T.L., Groves C.L., Smith D.L. (2021). Sensitive and Specific qPCR and Nested RT-PCR Assays for the Detection of Tobacco Streak Virus in Soybean. PhytoFrontiers.

[56] West-Ortiz M., Stuehler D., Pollock E., Wilson J.R., Preising S., Larrea-Sarmiento A., Alabi O., Fuchs M., Heck M., Olmedo-Velarde A. (2023). Characterization of cotton virus A, a novel and distinct member of the genus Caulimovirus with endogenous viral elements in *Gossypium* spp.. bioRxiv.

[57] Silva T.F., Romanel E.A., Andrade R.R., Farinelli L., Østerås M., Deluen C., Corrêa R.L., Schrago C.E., Vaslin M.F. (2011). Profile of Small Interfering RNAs from Cotton Plants Infected with the Polerovirus Cotton leafroll dwarf virus. BMC Mol. Biol..

[58] Kavalappara S.R., Bag S., Luckew A., McGregor C.E. (2023). Small RNA Profiling of Cucurbit Yellow Stunting Disorder Virus from Susceptible and Tolerant Squash (*Cucurbita pepo*) Lines. Viruses.

[59] Pecman A., Kutnjak D., Gutiérrez-Aguirre I., Adams I., Fox A., Boonham N., Ravnikar M. (2017). Next Generation Sequencing for Detection and Discovery of Plant Viruses and Viroids: Comparison of Two Approaches. Front. Microbiol..

[60] Goodstein D.M., Shu S., Howson R., Neupane R., Hayes R.D., Fazo J., Mitros T., Dirks W., Hellsten U., Putnam N. (2012). Phytozome: A Comparative Platform for Green Plant Genomics. Nucleic Acids Res..

[61] Yu J., Jung S., Cheng C.-H., Lee T., Zheng P., Buble K., Crabb J., Humann J., Hough H., Jones D. (2021). Cottongen: The Community Database for Cotton Genomics, Genetics, and Breeding Research. Plants.

[62] Xin M., Cao M., Liu W., Ren Y., Lu C., Wang X. (2017). The Genomic and Biological Characterization of Citrullus Lanatus Cryptic Virus Infecting Watermelon in China. Virus Res..

[63] Adeleke I.A., Kavalappara S.R., McGregor C., Srinivasan R., Bag S. (2022). Persistent, and Asymptomatic Viral Infections and Whitefly-Transmitted Viruses Impacting Cantaloupe and Watermelon in Georgia, USA. Viruses.

[64] Sedhain N.P., Bag S., Morgan K., Carter R., Triana P., Whitaker J., Kemerait R.C., Roberts P.M. (2021). Natural Host Range, Incidence on Overwintering Cotton and Diversity of Cotton Leafroll Dwarf Virus in Georgia USA. Crop Prot..

[65] Altschul S.F., Gish W., Miller W., Myers E.W., Lipman D.J. (1990). Basic Local Alignment Search Tool. J. Mol. Biol..

[66] Tamura K., Stecher G., Kumar S. (2021). MEGA11: Molecular Evolutionary Genetics Analysis Version 11. Mol. Biol. Evol..

[67] Randell T.M., Roberts P.M., Culpepper A.S. (2021). Palmer Amaranth (Amaranthaceae) and At-Plant Insecticide Impacts on Tarnished Plant Bug (Hemiptera: Miridae) and Injury to Seedling Cotton Terminals. J. Entomol. Sci..

[68] Shirley A. Thrips Management in Cotton. https://site.extension.uga.edu/tattnall/2023/04/thrips-management-in-cotton.

[69] Camp Hand G.H., Kemerait B., Liu Y., Perry C., Hall D., Mallard J., Porter W., Roberts P., Smith A., Virk S. (2023). Georgia Cotton Production Guide 2023.

[70] Kennedy G.G. Thrips Infestation Predictor for Cotton: An Online Tool for Informed Thrips Management. https://www.planthealthexchange.org/cotton/Pages/GROW-COT-04-17-103.aspx.

[71] Cook D., Herbert A., Akin D.S., Reed J. (2011). Biology, Crop Injury, and Management of Thrips (Thysanoptera: Thripidae) Infesting Cotton Seedlings in the United States. J. Integr. Pest Manag..

[72] Roberts P.M., Toews M. (2023). Commercial Insect and Weed Control in Cotton.

[73] Lockhart B.E., Menke J., Dahal G., Olszewski N.E. (2000). Characterization and Genomic Analysis of Tobacco Vein Clearing Virus, A Plant Pararetrovirus that is Transmitted Vertically and Related to Sequences Integrated in the Host Genome. J. Gen. Virol..

[74] Richert-Pöggeler K.R., Shepherd R.J. (1997). Petunia Vein-Clearing Virus: A Plant Pararetrovirus with the Core Sequences for an Integrase Function. Virology.

[75] Richert-Pöggeler K.R., Noreen F., Schwarzacher T., Harper G., Hohn T. (2003). Induction of Infectious Petunia Vein Clearing (Pararetro) Virus from Endogenous Provirus in Petunia. EMBO J..

[76] Harper G., Richert-Pöggeler K.R., Hohn T., Hull R. (2003). Detection of Petunia Vein-Clearing Virus: Model for the Detection of DNA Viruses in Plants with Homologous Endogenous Pararetrovirus Sequences. J. Virol. Methods.

[77] Eid S., Pappu H.R. (2014). Expression of Endogenous Para-Retroviral Genes and Molecular Analysis of the Integration Events in its Plant Host *Dahlia variabilis*. Virus Genes.

[78] Squires J., Gillespie T., Schoelz J.E., Palukaitis P. (2011). Excision and Episomal Replication of Cauliflower Mosaic Virus Integrated into a Plant Genome. Plant Physiol..

[79] Wang Y., Folimonova S.Y. (2023). Long Noncoding RNAs in Plant-Pathogen Interactions. Phytopathology.

[80] Mattick J.S., Amaral P.P., Carninci P., Carpenter S., Chang H.Y., Chen L.-L., Chen R., Dean C., Dinger M.E., Fitzgerald K.A. (2023). Long non-coding RNAs: Definitions, Functions, Challenges and Recommendations. Nat. Rev. Mol. Cell Biol..

[81] Nizamani M.M., Zhang Q., Muhae-Ud-Din G., Wang Y. (2023). High-throughput Sequencing in Plant Disease Management: A Comprehensive Review of Benefits, Challenges, and Future Perspectives. Phytopathol. Res..

[82] González-Pérez E., Chiquito-Almanza E., Villalobos-Reyes S., Canul-Ku J., Anaya-López J.L. (2024). Diagnosis and Characterization of Plant Viruses Using HTS to Support Virus Management and Tomato Breeding. Viruses.

[83] Ghoshal B., Sanfaçon H. (2015). Symptom Recovery in Virus-Infected Plants: Revisiting the Role of RNA Silencing Mechanisms. Virology.

[84] Schepetilnikov M., Ryabova L., Gaur R.K., Hohn T., Sharma P. (2014). Cauliflower Mosaic Virus (CaMV) Upregulates Translation Reinitiation of its Pregenomic Polycistronic 35S RNA via Interaction with the Cell’s Translation Machinery. Plant Virus–Host Interaction.

[85] Pahalawatta V., Druffel K., Pappu H.R. (2007). Seed Transmission of Dahlia mosaic virus in *Dahlia pinnata*. Plant Dis..

[86] Mette M.F., Kanno T., Aufsatz W., Jakowitsch J., van der Winden J., Matzke M.A., Matzke A.J. (2002). Endogenous Viral Sequences and their Potential Contribution to Heritable Virus Resistance in Plants. EMBO J..

[87] Bertsch C., Beuve M., Dolja V.V., Wirth M., Pelsy F., Herrbach E., Lemaire O. (2009). Retention of the Virus-Derived Sequences In the Nuclear Genome of Grapevine as a Potential Pathway to Virus Resistance. Biol. Direct.

[88] Aswad A., Katzourakis A. (2012). Paleovirology and Virally Derived Immunity. Trends Ecol. Evol..

[89] Valli A.A., Gonzalo-Magro I., Sanchez D.H. (2023). Rearranged Endogenized Plant Pararetroviruses as Evidence of Heritable RNA-based Immunity. Mol. Biol. Evol..

