# Supplementary material for: Characterization of Caulimovirid-like Sequences from Upland Cotton (Gossypium hirsutum L.) Exhibiting Terminal Abortion in Georgia, USA
Source: Viruses. 2024 Jul 11;16(7):1111. doi: 10.3390/v16071111 (PMC11281623; doi:10.3390/v16071111)
Supplement: Supplementary file 1 [file viruses-16-01111-s001.zip › viruses-3094186-supplementary.pdf]

Supplementary data:

Table S1: ***In silico*** **mining of integrated caulimovirid-like EVE sequences in the various** ***Gossypium*** **sps. using cotton databases**

Location	Species	Annotation version	E-value	% identity	Align length	Strands	Query from	Query to	Target from	Target to
**D04**	*G.barbadense*	v1.1	0	81	953	+/+	4898	5849	2028496	2029447
**D04**	*G.barbadense*	v1.1	0	75	1099	+/+	1336	2431	2026877	2027954
**D04**	*G.darwinii*	v1.1	0	75	1462	+/+	4632	6083	2168902	2170343
**D04**	*G.darwinii*	v1.1	0	80	953	+/+	4898	5849	2181416	2182367
**A03**	*G.tomentosum*	v1.1	0	79	5341	+/+	798	6094	102007327	102012565
**D04**	*G.tomentosum*	v1.1	0	81	953	+/+	4898	5849	2140473	2141421
**D04**	*G.tomentosum*	v1.1	0	76	1099	+/+	1336	2431	2138863	2139937
**A13**	*G.tomentosum*	v1.1	0	75	1112	+/-	4961	6063	103755520	103754420
**A04**	*G.hirsutum_Coker*	v1.1	0	100	7042	+/-	1	7042	80638708	80631669
**A04**	*G.hirsutum_Coker*	v1.1	0	99	6229	+/+	1	6225	80623669	80629889
**A04**	*G.hirsutum_Coker*	v1.1	0	97	394	+/+	7025	7413	80633655	80634048
**A04**	*G.hirsutum_FM958*	v1.1	0	100	7042	+/-	1	7042	80682226	80675187
**A04**	*G.hirsutum_FM958*	v1.1	0	99	6229	+/+	1	6225	80667187	80673407
**A04**	*G.hirsutum_FM958*	v1.1	0	97	394	+/+	7025	7413	80677173	80677566
**A04**	*G.hirsutum_DeltaPearl*	v1.1	0	100	7042	+/-	1	7042	80667859	80660820
**A04**	*G.hirsutum_DeltaPearl*	v1.1	0	99	6229	+/+	1	6225	80652820	80659040
**A04**	*G.hirsutum_DeltaPearl*	v1.1	0	97	394	+/+	7025	7413	80662806	80663199
**A04**	*G.hirsutum*	v3.1	0	100	7042	+/-	1	7042	80045055	80038016
**A04**	*G.hirsutum*	v3.1	0	99	6229	+/+	1	6225	80030016	80036236
**A04**	*G.hirsutum*	v3.1	0	97	394	+/+	7025	7413	80040002	80040395
**A04**	*G.hirsutum_CSX8308*	v1.1	0	100	7042	+/-	1	7042	80355540	80348501
**A04**	*G.hirsutum_CSX8308*	v1.1	0	99	6229	+/+	1	6225	80340501	80346721
**A04**	*G.hirsutum_CSX8308*	v1.1	0	97	394	+/+	7025	7413	80350487	80350880
**A04**	*G.hirsutum_UA48*	v1.1	0	100	7042	+/-	1	7042	79992710	79985671

**A04**	*G.hirsutum_UA48*	v1.1	0	99	6229	+/+	1	6225	79977671	79983891
**A04**	*G.hirsutum_UA48*	v1.1	0	97	394	+/+	7025	7413	79987657	79988050
**A04**	*G.hirsutum_UGA230*	v1.1	0	100	7042	+/-	1	7042	79933039	79926000
**A04**	*G.hirsutum_UGA230*	v1.1	0	99	6229	+/+	1	6225	79918000	79924220
**A04**	*G.hirsutum_UGA230*	v1.1	0	97	394	+/+	7025	7413	79927986	79928379
**A04**	*G.hirsutum*	v2.1	0	100	7042	+/-	1	7042	80620206	80613167
**A04**	*G.hirsutum*	v2.1	0	99	6229	+/+	1	6225	80605168	80611387
**A04**	*G.hirsutum*	v2.1	0	97	394	+/+	7025	7413	80615153	80615546
**A04**	*G.hirsutum*	v1.1	0	100	7042	+/-	1	7042	74680136	74673097
**A04**	*G.hirsutum*	v1.1	0	99	6229	+/+	1	6225	74665098	74671317
**A04**	*G.hirsutum*	v1.1	0	97	394	+/+	7025	7413	74675083	74675476
**scaffold_590**	*G.hirsutum*	v3.1	0	99	7059	+/+	1	7042	21796	28823
**D03**	*G.hirsutum_Coker*	v1.1	0	84	4080	+/+	1	4048	4110884	4114884
**D03**	*G.hirsutum*	v3.1	0	84	4080	+/+	1	4048	4064996	4068996
**D03**	*G.hirsutum_UA48*	v1.1	0	84	4080	+/+	1	4048	4091889	4095889
**D03**	*G.hirsutum*	v2.1	0	84	4080	+/+	1	4048	4160650	4164650
**D03**	*G.hirsutum*	v1.1	0	84	4080	+/+	1	4048	4240736	4244736
**D03**	*G.hirsutum_FM958*	v1.1	0	84	4080	+/+	1	4048	4073383	4077383
**D03**	*G.hirsutum_DeltaPearl*	v1.1	0	84	4080	+/+	1	4048	4065268	4069268
**D03**	*G.hirsutum_CSX8308*	v1.1	0	84	4080	+/+	1	4048	4130947	4134947
**D03**	*G.hirsutum_UGA230*	v1.1	0	84	4080	+/+	1	4048	4137270	4141270
**A05**	*G.hirsutum_Coker*	v1.1	0	73	3037	+/+	1422	4363	59981679	59984677
**A05**	*G.hirsutum_FM958*	v1.1	0	73	3037	+/+	1422	4363	59850004	59853002
**A05**	*G.hirsutum_DeltaPearl*	v1.1	0	73	3037	+/+	1422	4363	59908995	59911993
**A05**	*G.hirsutum*	v3.1	0	73	3037	+/+	1422	4363	59778505	59781503
**A05**	*G.hirsutum_CSX8308*	v1.1	0	73	3037	+/+	1422	4363	59762457	59765455
**A05**	*G.hirsutum_UA48*	v1.1	0	73	3037	+/+	1422	4363	59337644	59340642
**A05**	*G.hirsutum_UGA230*	v1.1	0	73	3037	+/+	1422	4363	59691548	59694546
**A05**	*G.hirsutum*	v2.1	0	73	3037	+/+	1422	4363	60033890	60036888
**scaffold_134**	*G.hirsutum*	v1.1	0	73	3037	+/+	1422	4363	40325	43323

**D07**	*G.hirsutum_Coker*	v1.1	0	80	1059	+/-	5044	6097	2848709	2847651
**D07**	*G.hirsutum_FM958*	v1.1	0	80	1059	+/-	5044	6097	2815839	2814781
**D07**	*G.hirsutum_DeltaPearl*	v1.1	0	80	1059	+/-	5044	6097	2806525	2805467
**D07**	*G.hirsutum*	v3.1	0	80	1059	+/-	5044	6097	2822754	2821696
**D07**	*G.hirsutum_CSX8308*	v1.1	0	80	1059	+/-	5044	6097	2814226	2813168
**D07**	*G.hirsutum_UA48*	v1.1	0	80	1059	+/-	5044	6097	2658605	2657547
**D07**	*G.hirsutum_UGA230*	v1.1	0	80	1059	+/-	5044	6097	2843521	2842463
**D07**	*G.hirsutum*	v2.1	0	80	1059	+/-	5044	6097	2848951	2847893
**D07**	*G.hirsutum*	v1.1	0	80	1059	+/-	5044	6097	2843658	2842600
**A13**	*G.hirsutum_Coker*	v1.1	0	76	1254	+/-	4821	6065	105253892	105252652
**A13**	*G.hirsutum*	v3.1	0	76	1254	+/-	4821	6065	104414249	104413009
**A13**	*G.hirsutum*	v2.1	0	76	1254	+/-	4821	6065	104661058	104659818
**A13**	*G.hirsutum*	v1.1	0	76	1254	+/-	4821	6065	97601618	97600378
**A13**	*G.hirsutum_CSX8308*	v1.1	0	76	1254	+/-	4821	6065	104162913	104161673
**D07**	*G.mustelinum*	v1.1	0	70	5134	+/+	991	6065	615272	620222
**D07**	*G.mustelinum*	v1.1	0	71	3254	+/-	2847	6065	628092	624899
**D07**	*G.mustelinum*	v1.1	0	73	2113	+/+	3970	6065	628483	630565
**D07**	*G.mustelinum*	v1.1	0	72	1319	+/-	4754	6065	641766	640459
**D05**	*G.mustelinum*	v1.1	0	78	1333	+/+	4755	6081	7182119	7183438
**A07**	*G.mustelinum*	v1.1	0	75	1527	+/+	4555	6065	391111	392611
**A04**	*G. stephensii*	v1.0	0	99	7042	+/+	1	7042	79018976	79011937
**A04**	*G. stephensii*	v1.0	0	99	6225	+/+	1	6225	79018976	79011937
**D03**	*G. stephensii*	v1.0	0	84	3686	+/+	35	3702	79003935	79010156
**D07**	*G. stephensii*	v1.0	0	80	1045	+/+	5044	6084	2841911	2840867

Supplementary Figures:

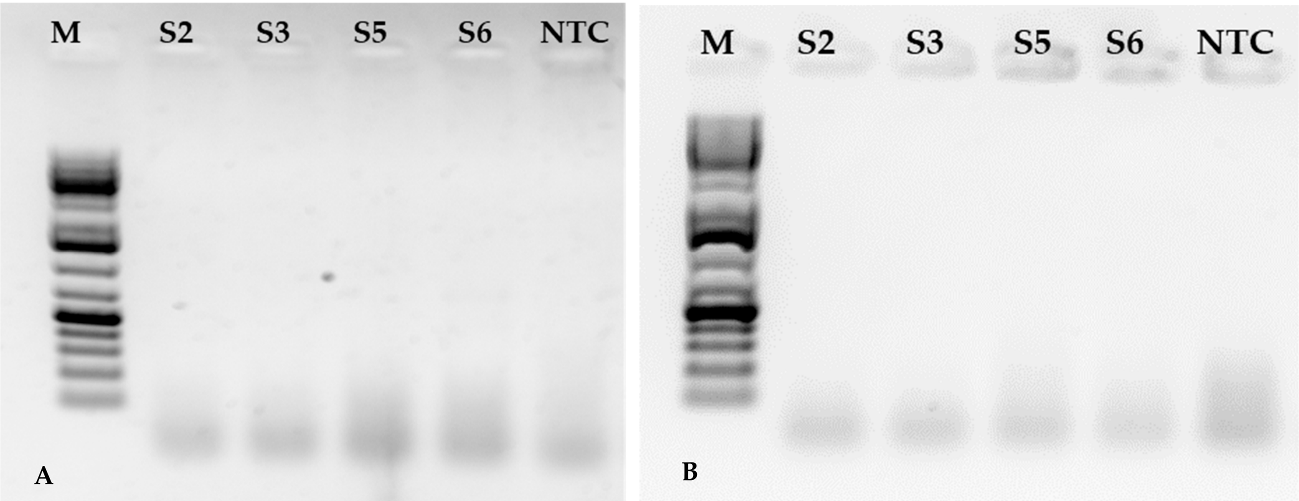

Figure S1: Detection of tobacco steak virus (TSV) using RT-PCR, (**A**) coat protein gene using primer pair SB162 F/R and (**B**) movement protein gene using primer pair SB164 F/R; M- Marker, Samples: S2, S3, S5, S6, NTC-No template control.

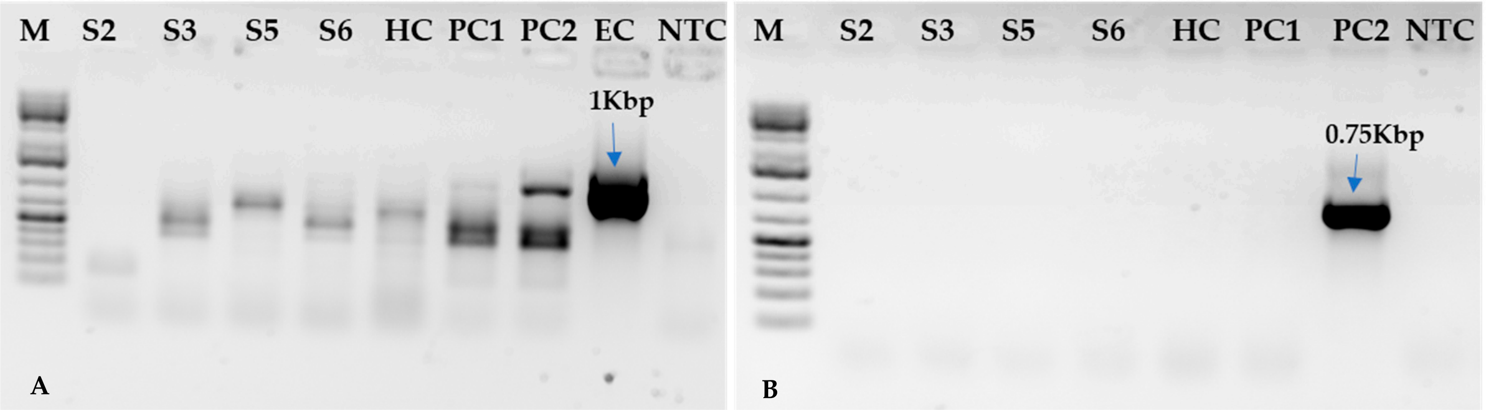

Figure S2: Detection of cotton leafroll dwarf virus (CLRDV) using RT-PCR, (**A**) P0 protein gene using primer pair SB28 F/R; (**B**) Coat protein gene detection using primer pair SB11F/R; Lanes were marked as: M-Marker; Samples: S2, S3, S5, S6; HC-Healthy control; PC1 and PC2- plant positive control; EC- P0 amplicons eluted from prior PCR used as positive control for P0 gene, NTC-No template control
